# Identifying clusters of healthcare expenditure trajectories in end-stage organ disease: a retrospective cohort study using linked administrative databases in Singapore

**DOI:** 10.1186/s12913-025-13590-z

**Published:** 2025-10-22

**Authors:** Sheryl Hui-Xian Ng, Palvinder Kaur, Laurence Lean Chin Tan, Ri Yin Tay, Mervyn Yong Hwang Koh, Andy Hau Yan Ho, Allyn Hum, Woan Shin Tan

**Affiliations:** 1 Health Services and Outcomes Research, National Healthcare Group, 3 Fusionopolis Link, #03-08, Singapore, 138543 Singapore; 2 Department of Geriatric Medicine, Khoo Teck Puat Hospital, Singapore, Singapore; 3 Dover Park Hospice, Singapore, Singapore; 4 Psychology, School of Social Sciences, Nanyang Technological University, Singapore, Singapore; 5 Lee Kong Chian School of Medicine, Nanyang Technological University, Singapore, Singapore; 6 The Palliative Care Centre for Excellence in Research and Education, Singapore, Singapore; 7 Department of Palliative Medicine, Tan Tock Seng Hospital, Singapore, Singapore; 8 Geriatric Education & Research Institute, Singapore, Singapore

**Keywords:** Healthcare expenditure, End-stage organ disease, Clustering

## Abstract

**Objectives:**

We aimed to identify subgroups of decedents with end-stage organ disease based on their healthcare expenditure trajectories over the final five years of life, and factors associated with incurring high costs in this period.

**Methods:**

We conducted a retrospective cohort study of patients who died between 2017 and 2019, who had either a primary or secondary diagnosis of advanced dementia, severe liver disease, as well as heart, kidney or respiratory failure in the last five years of their lives. Data was extracted from administrative databases of a regional health system in Singapore. We classified patients into subgroups with distinctly different five-year healthcare expenditure trajectories, using longitudinal k-means clustering. Factors associated with membership in each subgroup were then identified through multinomial modelling.

**Results:**

Among 7,154 decedents, three trajectories of consistently low cost (LC, *n* = 5,756), moderately high cost near death (MC, *n* = 1,283) and escalating cost near death (EC, *n* = 115) were identified. Patients with MC often had concurrent end-stage organ diseases and chronic conditions, while patients with EC were younger and often had respiratory failure. Across all subgroups, most patients had at least one emergency department attendance in their final three months and either late or no access to palliative care. Accounting for socio-demographic characteristics and comorbidity, patients with respiratory failure were more likely to have a high-cost trajectory.

**Conclusions:**

Our findings highlight the significance of upstream interventions to address needs of multi-morbid older adults, and pertinence of timely palliative care access to mitigate the use of aggressive care at the end-of-life.

**Supplementary information:**

The online version contains supplementary material available at 10.1186/s12913-025-13590-z.

## Introduction

Patients with end-stage organ disease (ESOD) live with significant physical and psychological symptoms comparable to that experienced by patients with advanced cancer [1]. Despite the similarity in symptom burden, patients with ESOD have comparatively poorer access to palliative care [2, 3]. Due to difficulties faced in prognostication, identifying patients for timely palliative referral is challenging. Patients with ESOD are hence likely to experience an unpredictable trajectory of intermittent exacerbations and recovery over years before death [4], leading to high end-of-life (EOL) costs within the final year before death [5, 6]. As these high healthcare costs may also signal potential inefficiencies in current care processes [7], developing a thorough understanding of patients with ESOD and their cost burden and drivers at the EOL would be a pertinent prelude to the development of strategies to concurrently mitigate high EOL costs whilst improving quality of care for patients in their final months.

However, with the high heterogeneity of disease aetiology and progression within ESOD, profiling these patients with a one-size-fits-all approach may not provide actionable insight. Furthermore, a prior study of EOL healthcare costs associated with ESODs highlighted that these patients often had multiple conditions [8], inhibiting effective intervention from a disease-specific perspective. As an approach to accommodate these unique features of ESOD, segmenting patients into smaller subgroups could facilitate the design and organisation of interventions around the characteristics and needs of each subgroup [9]. For instance, in a study of cancer patients referred for home-based palliative care, Zhuang et al. classified patients into one of three segments based on their hospital utilisation patterns after referral, pointing to the different palliative interventions relevant to meet the needs of each segment [10].

Further, specific to addressing high EOL costs, several studies have segmented patients by clustering their healthcare cost trajectories, spotlighting subgroups with the propensity to incur high healthcare costs over time. Within the final year of life, Von Wyl et al. identified five subgroups of trajectories among older decedents, with 6% of patients incurring the most expenditure with a steady increase over time and a drop in the final month [11]. Davis et al. identified four trajectories among Medicare decedents, with 49% incurring high persistent spending over the months leading to death [12]. Looking beyond the final year, Hansen et al. identified four spending trajectories among Danish decedents, with 86% incurring consistently moderate or high trajectories [13], while Teraoka et al. similarly identified six trajectories among Japanese decedents, with 46% of patients in the high-persistent trajectory group [14]. Consistently across these studies, one or more subgroups have been identified to incur persistently high costs over time, warranting further exploration for intervention.

Nonetheless, despite the demonstrated utility in elderly decedent populations, to date, population segmentation approaches have not been applied to patients with ESOD. Segmentation of an ESOD patient cohort through the clustering of cost trajectories could identify subgroups with different trajectories over multiple years before death, facilitating intervention prior to the final year. Comparing and contrasting the subgroups can provide insight into potential drivers of high costs, and approaches and junctures for palliative intervention specific to each group can be identified. Hence, the aim of this study is to identify subgroups of patients with ESOD based on their healthcare expenditure trajectories over five years prior to death, profile each subgroup, and identify factors associated with high cost. Findings will generate evidence for subsequent policy measures to mitigate cost increase and inform timely development of interventions to impact clinical care.

## Methods

### Setting

In Singapore, more than 20,000 people die per year, with more than half of them dying from cancer, pneumonia or ischemic heart diseases [15]. People with these and other life-limiting conditions can be referred by palliative specialists in the public healthcare system to receive care in inpatient, community or home hospices, often subsidised by the government. However, uptake of these services has been notably late at less than one month before death [16], suggesting that most of EOL care remains centred within the hospitals.

The public healthcare system in Singapore is organised into three regional health systems (RHS), each consisting of a network of acute and community hospitals, national specialty centres and primary care clinics [17]. The National Healthcare Group (NHG) RHS serves as the healthcare manager of residents residing in the Central and Northern regions of Singapore, overseeing the health of more than 1.5 million, or a quarter of Singapore’s population [18, 19]. This study is sited with Tan Tock Seng Hospital (TTSH) and Khoo Teck Puat Hospital (KTPH), which are acute hospitals within the NHG RHS that house dedicated beds for delivery of palliative care.

### Study design and population

This was a retrospective cohort study, with inclusion criteria and data sources described in detail elsewhere [8]. In brief, all patients who died between January 2017 and December 2019, aged more than 21 years old, and diagnosed with any ESOD in TTSH or KTPH in the last five years of their lives were included. Patients who had an advanced cancer diagnosis were excluded. ESODs included in this study comprised heart failure (HF), respiratory failure (RF), kidney failure (KF), severe liver disease (SLD), and advanced dementia (AD) (Additional File 1 and 2).

### Data source and variables

Data was extracted from the NHG RHS database, a data warehouse consisting of episodic data within the health system [20]. We extracted information on patients’ date of death from the national death registry, as well as visits to the inpatient, specialist outpatient, emergency department (ED) and day procedure (DP) settings of TTSH and KTPH up to five years before death. Healthcare expenditure (HCE) was measured by summing the gross charge, which reflects the cost to the patient prior to receipt of government subsidies. The HCE reported in this study was deflated by the Consumer Price Index to the base year 2019 [21], and converted to British pounds (1 SGD = 0.59 GBP). Additionally, as a proxy of intensity of care provided at the EOL, we reported data on ED attendance, intensive care or high dependency unit admission, as well as endotracheal intubation in the final three months of life [22]. As palliative consultations within the hospitals are often patients’ first point of access to palliative services both within and beyond the hospital, to establish the level of access to palliative care in this cohort, referrals for palliative consultations for each patient were also retrieved from institutional databases maintained by the palliative teams in TTSH and KTPH.

In addition to visit information, we extracted information on patients’ socio-demographic characteristics, duration of ESOD diagnosis, multi-morbidity of ESOD, chronic condition diagnosis. Patients were categorised as ethnically Chinese or non-Chinese, as approximately 75% of the resident population in Singapore are of Chinese descent [23], and the remaining minority are of Malay, Indian and other ethnicities. Also, as more than 75% of residents live in public housing [24], and average household income increases with apartment size, housing type was reported as a proxy of patients’ socio-economic status. As patients could have more than one ESOD diagnosis, they were counted in multiple groups if they had more than one condition. Multi-morbidity was then reflected by the number of ESOD diagnoses a patient was recorded to have within the last five years of life. Chronic conditions were shortlisted from the list monitored by the Ministry of Health in Singapore under the Chronic Disease Management Programme [25].

### Statistical methodology

We applied longitudinal k-means estimation to cluster patients’ five-year HCE trajectories into subgroups, using the *kml* package in R [26]. For each patient, their HCE was summed for each quarter, or three-month interval, prior to their death date. As patients may either have no HCE for a particular quarter over the five-year period, or conversely incur extremely high costs, the resultant HCE data may be highly skewed with several zeroes, which would be challenging to fit to a suitable statistical model [27]. In contrast, k-means estimation, a non-parametric approach, would not be constrained by the statistical distribution of the data, and has been demonstrated for use in studies clustering healthcare claims data [28, 29].

To identify the number of subgroups with the best performance in maximizing both between-group differences and within-group similarities, we split patients into 2–7 subgroups [30], and assessed the performance of the clustering each time using criterion scores, such as the Calinski Harabatz, Ray Turie and David Bouldin indices [26]. We assessed stability of our approach by repeating each clustering process for 50 iterations with different starting values. In addition, we validated our findings by repeating the process on 50 bootstrap resamples of the data and reporting the distribution of the clustering results. To account for the impact of including patients who presented for the first time close to death, sensitivity analyses excluding patients who were diagnosed up to 30 days before death were conducted.

With the optimal number of subgroups identified, we described the HCE trajectory for each subgroup. The mean HCE over the last three months, one year and five years for each subgroup were reported. Each subgroup was then profiled across the domains of socio-demographic characteristics, ESOD diagnosis, chronic condition prevalence, as well as end-of-life care received. Fisher’s tests and Wilcoxon rank-sum tests were used for between-group comparisons of categorical and continuous variables respectively.

To quantify the strength of association between patient characteristics and the propensity to be a high-cost patient, multinomial logistic regression was used. The probability of being in each high-cost subgroup was modelled, using the domains of patient characteristics described in the profiling. As several statistical tests were performed in the process of between-group comparisons, we accounted for potential multiple testing with a Bonferroni correction [31], resulting in a p-value threshold of 0.001. All analyses were conducted in RStudio version 2023.03.0. [32].

## Results

Of 7,154 patients with an ESOD diagnosis (Additional File 3), we identified three groups of five-year HCE trajectories prior to death, this approach produced the highest and most stable criterion scores (Additional File 4, Fig. 1). The first subgroup, representing 5,756 patients (80.5%), had a trajectory of consistently low cost (LC) throughout the five-year period. The second subgroup, representing 1,283 patients (17.9%), had a trajectory of moderately high cost near death (MC). The third subgroup, representing 115 patients (1.6%), had a trajectory of escalating cost near death (EC). Repeating the clustering process over 50 bootstrap iterations and classifying patients into three subgroups had the best criterion performance in more than half the iterations (2 subgroups: 11, 22.0%; 3 subgroups: 27, 54.0%; 4 subgroups: 12, 24.0%). Sensitivity analyses repeating the clustering after excluding patients diagnosed with ESOD in the last month of life retained the same three groups among the remaining 6,292 patients (88.0%) (Additional File 5), where only 17 shifted in their group membership.

**Fig. 1:**
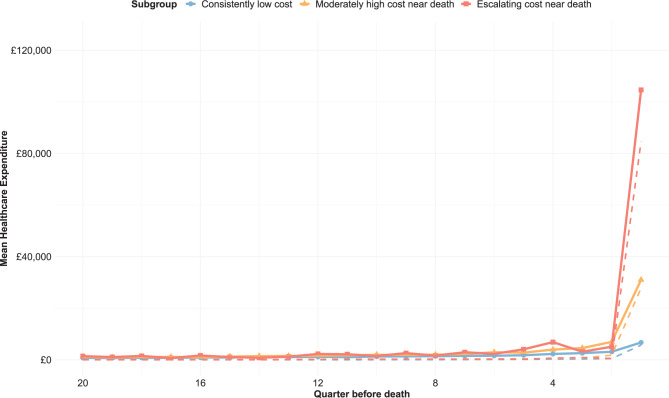
Healthcare expenditure trajectories over the final five years of life among patients with end-stage organ disease, by subgroup. Solid lines reflect mean quarterly healthcare expenditure for each subgroup, while dashed lines reflect median values

### Profiles of each subgroup

Patient profiles of the three subgroups are described in Table 1. Patients had distinctly different socio-demographic characteristics. While the median age of patients in LC was 83 years, the median age was significantly lower at 77 and 70 years respectively for patients with MC and EC (both *p* < 0.001). The proportions of patients who were male or of ethnic minority were significantly higher among patients with MC (male sex: LC: 49.1%; MC: 58.4%, *p* < 0.001; EC: 65.2%, *p* ≥ 0.001; ethnic minority: LC: 24.3%; MC: 29.2%, *p* < 0.001; EC: 34.8%, *p* ≥ 0.001). A significantly larger proportion of patients with MC resided in 1–2-room public housing compared to patients with LC, but there was no significant difference with patients with EC (LC: 5.8%; MC: 7.3%, *p* < 0.001; EC: 12.2%, *p* ≥ 0.001). While patients with LC and EC were similar in prevalence of frequently occurring chronic conditions, patients with MC had a higher prevalence of hypertension, dyslipidaemia, diabetes and stroke (all *p* < 0.001) (Table 1).

**Table 1:** Profiles of subgroups identified

		Consistently low-cost (n = 5,756, 80%)	Moderately high cost near death (n = 1,283, 18%)		Escalating cost near death (n = 115, 2%)	
**Age**	Median (25^th^ − 75^th^ percentile)	83 (74–89)	77 (67–83.5)	***	70 (58–79)	***
**Age category**	Below 65 years	602 (10.5%)	243 (18.9%)	***	44 (38.3%)	***
	65–74 years	870 (15.1%)	339 (26.4%)		35 (30.4%)	
	75–84 years	1,681 (29.2%)	429 (33.4%)		26 (22.6%)	
	85 years and above	2,603 (45.2%)	272 (21.2%)		10 (8.7%)	
**Sex**	Female	2,928 (50.9%)	534 (41.6%)	***	40 (34.8%)	NS
	Male	2,828 (49.1%)	749 (58.4%)		75 (65.2%)	
**Ethnicity**	Majority	4,358 (75.7%)	909 (70.8%)	***	75 (65.2%)	NS
	Minority	1,398 (24.3%)	374 (29.2%)		40 (34.8%)	
**Residence type**	Public housing, 1–2-room	334 (5.8%)	94 (7.3%)	***	14 (12.2%)	NS
	Public housing, 3–4-room	2,502 (43.5%)	627 (48.9%)		49 (42.6%)	
	Public housing, 5-room and larger	969 (16.8%)	216 (16.8%)		19 (16.5%)	
	Residential nursing home	1,226 (21.3%)	204 (15.9%)		21 (18.3%)	
	Private or other housing	725 (12.6%)	142 (11.1%)		12 (10.4%)	
**Number of ESOD diagnoses over 5 years**	1	3,959 (68.8%)	646 (50.4%)	***	63 (54.8%)	NS
	2	1,365 (23.7%)	431 (33.6%)		31 (27.0%)	
	3	377 (6.5%)	180 (14.0%)		19 (16.5%)	
	4	55 (1.0%)	26 (2.0%)		2 (1.7%)	
**ESOD diagnosis over 5 years**						
Advanced dementia		2,576 (44.8%)	381 (29.7%)	***	13 (11.3%)	***
Kidney failure		1,587 (27.6%)	437 (34.1%)	***	50 (43.5%)	***
Heart failure		1,810 (31.4%)	579 (45.1%)	***	46 (40.0%)	NS
Respiratory failure		1,457 (25.3%)	553 (43.1%)	***	65 (56.5%)	***
Severe liver disease		610 (10.6%)	202 (15.7%)	***	16 (13.9%)	NS
**Years from first ESOD diagnosis to death**	Median (25^th^ − 75^th^ percentile)	1.4 (0.4–3.2)	1.6 (0.3–3.6)	**NS**	1.2 (0.4–4.1)	**NS**
**Chronic conditions**						
Hypertension		4,749 (82.5%)	1,130 (88.1%)	***	90 (78.3%)	NS
Dyslipidaemia		3,151 (54.7%)	857 (66.8%)	***	66 (57.4%)	NS
Diabetes		3,125 (54.3%)	842 (65.6%)	***	69 (60.0%)	NS
Stroke		1,604 (27.9%)	442 (34.5%)	***	37 (32.2%)	NS
Osteoarthritis		771 (13.4%)	184 (14.3%)	NS	11 (9.6%)	NS
Benign prostrate hyperplasia		722 (12.5%)	205 (16.0%)	NS	12 (10.4%)	NS
Osteoporosis		620 (10.8%)	128 (10.0%)	NS	3 (2.6%)	NS
Mental conditions^		464 (8.1%)	119 (9.3%)	NS	7 (6.1%)	NS
Gout		438 (7.6%)	133 (10.4%)	NS	7 (6.1%)	NS
Parkinson’s disease		452 (7.9%)	82 (6.4%)	NS	5 (4.3%)	NS
Asthma		255 (4.4%)	83 (6.5%)	NS	6 (5.2%)	NS

ESOD: end-stage organ disease; ***: *p* < 0.001; NS: *p* ≥ 0.001; ^includes anxiety, depression, schizophrenia or bipolar disorder

In addition, the three subgroups differed in their composition of ESODs. Almost half of patients with LC had a diagnosis of AD in the five years prior to death, which was significantly higher than the other two subgroups (LC: 44.8%; MC: 29.7%; EC: 11.3%, both *p* < 0.001). Among patients with MC, almost half had a diagnosis of HF or RF, which was significantly more frequent than among patients with LC (LC, HF: 31.4%; RF: 25.3%; MC, HF: 45.1%, RF: 43.1%, both *p* < 0.001). Among patients with EC, more than half had RF, and four in ten had KF (RF: 56.5%, KF: 43.5%), which was also significantly more frequent than among patients with LC (both *p* < 0.001). While the duration between first ESOD diagnosis and death did not differ between the subgroups, the proportion of patients with multiple ESODs was significantly higher in MC than in LC (LC: 31.2%; MC: 49.6%, *p* < 0.001; EC: 45.2%, *p* ≥ 0.001).

### Healthcare expenditure and utilisation for each subgroup

In the final three months of life, patients with EC incurred the highest HCE (mean [standard deviation (SD)]: £104,626 [£48,618]), followed by patients with MC and LC respectively (MC: £31,002 [£11,216]; LC: £6,715 [£5,433]). This trend held within the final month (mean: LC: £4,485, MC: £21,276, EC: £82,264; maximum: LC: £20,512, MC: £67,373, EC: £288,655), and persisted for one- and five-year costs (Table 2, Additional File 6 and 7). Examining care patterns, inpatient costs accounted for the bulk of the HCE in the final three months (LC: 78.6% [29.6%]; MC: 96.8% [3.9%]; EC: 99.4% [0.7%]). Patients with LC on average were admitted once in this period (1.3 [1.0] admissions; length of stay (LOS): 13.4 [12.4] days), while patients with MC and EC had on average two inpatient admissions with longer stays (MC: 2.4 [1.4] admissions, LOS: 50.2 [26.0] days; EC: 1.8 [0.9] admissions, LOS: 104.0 [57.3] days). At one and five years before death, patients across all groups recorded multiple emergency attendances, inpatients admissions and SOC visits, with the abovementioned patterns in relative resource use between the groups persisting (Additional File 8).

**Table 2:** Healthcare expenditure and utilisation in the final 3 months of life by subgroup

	Consistently low-cost (n = 5,756)	Moderately high cost near death (n = 1,283)	Escalating cost near death (n = 115)
**Healthcare expenditure**	Mean (SD)		
Mean HCE	£ 6,715 (£ 5,433)	£ 31,002 (£ 11,216)	£ 104,626 (£ 48,618)
Mean HCE by setting			
Inpatient admissions	£ 6,110 (£ 5,287)	£ 30,142 (£ 11,350)	£ 104,076 (£ 48,734)
ED attendances	£ 384 (£ 326)	£ 556 (£ 445)	£ 365 (£ 381)
SOC visits	£ 197 (£ 356)	£ 263 (£ 700)	£ 150 (£ 376)
Day procedures	£ 23 (£ 217)	£ 41 (£ 298)	£ 35 (£ 237)
% of HCE by setting			
Inpatient admissions	78.6% (29.6%)	96.8% (3.9%)	99.4% (0.7%)
ED attendances	10.7% (17.4%)	2.1% (1.9%)	0.4% (0.4%)
SOC visits	10.1% (24.1%)	1.0% (3.1%)	0.2% (0.5%)
Day procedures	0.6% (5.6%)	0.1% (1.1%)	0.03% (0.2%)
**Healthcare utilisation**	Mean (SD)		
Number of inpatient admissions	1.3 (1.0)	2.4 (1.4)	1.8 (0.9)
Admission to intensive care unit	0.1 (0.3)	0.4 (0.5)	0.9 (0.5)
Admission to high-dependency unit	0.1 (0.3)	0.4 (0.6)	0.9 (0.6)
Inpatient length of stay (days)	13.4 (12.4)	50.2 (26.0)	104.0 (57.3)
Number of ED attendances	1.4 (1.1)	2.1 (1.5)	1.3 (1.1)
Number of SOC visits	2.3 (3.6)	2.9 (3.8)	1.7 (4.1)
Number of day procedures	0.03 (0.20)	0.04 (0.23)	0.03 (0.18)

ED: Emergency Department; HCE: healthcare expenditure; SD: standard deviation; SOC: specialist outpatient clinic

A larger proportion of patients with MC or EC received EOL care of high acuity, compared to patients with LC. Most patients across all subgroups had at least one ED attendance in the final three months of life, with the highest rate in patients with MC (92.4%) (Table 3). Compared to patients with LC, a larger proportion of patients with MC or EC were admitted to intensive or high dependency care - notably almost all patients with EC were admitted (LC: 12.2%; MC: 51.0%; EC: 89.6%, both *p* < 0.001). More than half of patients with EC were also intubated in the same period (LC: 5.0%; MC: 17.6%; EC: 51.3%). While the rate of palliative referral was higher in patients with MC and EC (LC: 21.2%; MC: 37.6%; EC: 49.6%; both *p* < 0.001), timing of referrals remained late across all subgroups, occurring within weeks before death (LC: median = 15 days; MC: 9 days; EC: 12 days; both *p* ≥ 0.001).

**Table 3:** Indicators on intensity of medical care provided at the end-of-life

		Consistently low-cost (n = 5,756, 80%)	Moderately high cost near death (n = 1,283, 18%)		Escalating cost near death (n = 115, 2%)	
**Acute care in final 3 months of life**						
At least 1 Emergency Department attendance		4,758 (82.7%)	1,185 (92.4%)	***	85 (73.9%)	NS
At least 1 intensive care/high dependency unit admission		705 (12.2%)	654 (51.0%)	***	103 (89.6%)	***
Received endotracheal intubation		285 (5.0%)	226 (17.6%)	***	59 (51.3%)	***
**Referral to palliative care**						
Ever referred		1,219 (21.2%)	482 (37.6%)	***	57 (49.6%)	***
By timing of referral	< 1 month before death	730 (12.7%)	336 (26.2%)	***	36 (31.3%)	NS
	1 to < 3 months before death	149 (2.6%)	68 (5.3%)		5 (4.3%)	
	3 to < 12 months before death	215 (3.7%)	50 (3.9%)		12 (10.4%)	
	≥1 year before death	125 (2.2%)	28 (2.2%)		4 (3.5%)	
	Not referred	4537 (78.8%)	801 (62.4%)		58 (50.4%)	
Days from first known referral to death	Median (25^th^ − 75^th^ percentile)	15.0 (3.0–114.5)	9.0 (3.0–40.8)	NS	12.0 (4.0–101.0)	NS

***: *p* < 0.001; NS: *p* ≥ 0.001

### Factors associated with cost trajectories

Accounting for socio-demographic characteristics, number of ESODs diagnosed and diagnosis of chronic conditions, patients with a diagnosis of RF were more likely to have a trajectory of MC or EC (Odds ratio (OR), 99.9% confidence interval (CI): MC: 1.65, 1.30–2.10; EC: 2.59, 1.29–5.21) (Table 4). Adjusting for the abovementioned factors, being aged 75 and older was associated with a lower likelihood (MC, 75–84 years: OR, 99.9% CI = 0.57, 0.40–0.81; ≥ 85 years: 0.27, 0.18–0.40; EC, 75–84 years: 0.28, 0.11–0.71; ≥ 85 years: 0.10, 0.03–0.37). In addition, having multiple ESOD diagnoses, or a history of stroke was also associated with a higher likelihood of a MC trajectory (2 ESODs: OR, 95% CI = 1.44, 1.12–1.85; 3 ESODs: 1.67, 1.16–2.40; stroke: 1.47, 1.16–1.85).

**Table 4:** Associations between patient characteristics and likelihood of having a high-cost trajectory

		Likelihood of being a patient with MC	Likelihood of being a patient with EC
		**Odds ratio (99.9% CI)**	**Odds ratio (99.9% CI)**
**Age category**	Below 65 years	Reference	Reference
	65–74 years	0.88 (0.63–1.24)	0.58 (0.26–1.28)
	75–84 years	**0.57 (0.40–0.81)**	**0.28 (0.11–0.71)**
	85 years and above	**0.27 (0.18–0.40)**	**0.10 (0.03–0.37)**
**Sex**	Female	Reference	Reference
	Male	1.03 (0.80–1.31)	1.11 (0.54–2.25)
**Ethnicity**	Majority	Reference	Reference
	Minority	0.92 (0.72–1.18)	0.98 (0.49–1.96)
**Residence type**	Public housing, 1–2-room	0.97 (0.63–1.49)	1.58 (0.56–4.50)
	Public housing, 3–4-room	Reference	Reference
	Public housing, 5-room and larger	0.92 (0.68–1.25)	1.08 (0.43–2.69)
	Residential nursing home	0.78 (0.57–1.07)	1.29 (0.52–3.20)
	Private or other housing	0.98 (0.69–1.39)	1.16 (0.39–3.44)
**ESOD diagnosis over 5 years**			
Advanced dementia		0.92 (0.70–1.20)	0.39 (0.13–1.14)
Kidney failure		0.92 (0.72–1.19)	1.29 (0.60–2.76)
Heart failure		1.13 (0.89–1.45)	0.88 (0.41–1.88)
Respiratory failure		**1.65 (1.30–2.10)**	**2.59 (1.29–5.21)**
Severe liver disease		1.30 (0.96–1.74)	0.87 (0.36–2.12)
**Number of ESOD diagnoses over 5 years**	1	Reference	Reference
	2	**1.44 (1.12–1.85)**	1.23 (0.56–2.71)
	3	**1.67 (1.16–2.40)**	2.12 (0.76–5.97)
	4	1.37 (0.86–2.18)	1.60 (0.41–6.32)
**Chronic conditions**			
Hypertension		1.18 (0.81–1.70)	0.79 (0.29–2.14)
Dyslipidaemia		1.07 (0.76–1.51)	0.76 (0.27–2.15)
Diabetes		1.17 (0.84–1.63)	1.03 (0.37–2.89)
Stroke		**1.47 (1.16–1.85)**	1.77 (0.88–3.56)
Benign prostrate hyperplasia		1.30 (0.94–1.81)	0.98 (0.33–2.95)
Osteoporosis		1.12 (0.78–1.60)	0.39 (0.05–2.80)
Gout		1.17 (0.81–1.69)	0.72 (0.19–2.73)

CI: confidence interval; EC: Escalating cost near death; ESOD: end-stage organ disease; MC: moderately high cost near death; entries in bold are statistically significant at *p* < 0.001

## Discussion

We identified three distinct trajectories of HCE over five years prior to death among patients diagnosed with ESOD. Patients with a trajectory of low cost over the five-year period mostly had a single diagnosis of ESOD, often advanced dementia. Patients with a trajectory of moderately high cost near death often had concurrent ESODs and a high prevalence of chronic conditions. Patients with a trajectory of escalating cost near death were younger and were more likely to have had respiratory failure. Across all three groups, most patients had at least one ED attendance in the final three months and had either late or no access to palliative care.

We identified two subgroups with high cumulative costs over multiple years before death. The average HCE in the final year for patients with MC and EC exceeded the highest cost in a similar study of ESOD decedents [5]. Moreover, the five-year costs reported for patients with EC are comparable to those for groups with persistently high cost in Teraoka et al. and Hansen et al. [13, 14]. However, we did not observe a clear persistent high-cost trajectory that was prominent across studies of older decedents. We could attribute this potentially to the slew of disease management and integrated care initiatives implemented for patients with organ disease in Singapore, which saw some success in reducing hospitalisations while maintaining good quality of care [33–35], translating to a lack of persistently high-cost groups. Also, as studies noting persistent high-cost trajectories were conducted on older patient populations that included patients with non-ESOD conditions, such as stroke and psychiatric conditions, we could infer that the disparity in part could be due to differences in inclusion criteria. Aside from our prior study of disease-associated costs for the same ESOD cohort [8], there remains no studies on the long-term cost burden of ESOD, and our five-year estimates of HCE contribute novel insights on types of multi-year cost trajectories in ESOD.

We saw that patients with multiple ESODs were more likely to have a high-cost trajectory, consistent with observations elsewhere that multimorbidity was associated with high costs and resource use in the last months of life [36]. The provision of coordinated care to address the needs of multi-morbid older adults has been demonstrated to moderate high healthcare utilisation [37]. Pathways integrating care provision across medical specialities and professionals aim to provide these patients with care beyond a single-disease focus to provide patient-centred care [38, 39]. In context of ESOD, implementation of such care models, even years prior to the end-stage, could have a tangible impact on EOL costs downstream [12, 14]. Further, in this study, patients diagnosed with RF were more likely to have moderately or escalating-cost trajectories even after accounting for other patient characteristics, suggesting that their existing resource use patterns were likely to be driving costs. Integration of supportive care into standard nephrological care among patients with chronic kidney disease has shown potential to improve quality of life, and reduce acute care costs [40]. Exploration of similar care models integrating care planning and early palliative care for patients with RF can be explored to meet their needs and temper use of burdensome interventions in the last weeks of life.

Increasing access to care planning and palliative care (PC) for patients with ESOD could address both issues of high EOL costs and use of aggressive care in the last months of life. We saw a large proportion of patients across all subgroups with ED attendances in the final three months of life, and more than half of patients with MC or EC requiring intensive or high dependency care. Coupled with low and late access to PC across all subgroups, our findings suggest a potential lack of goals-of-care conversations and default to curative care even in the final days in this patient cohort. As these conversations, which can include expressions of treatment and place-of-death preferences, can occur outside of a palliative referral, ensuring patients have an avenue to engage in prior care planning can support them to avoid unwanted treatment and costs in their final days [41]. In Singapore, information and tools related to the EOL and advance care planning (ACP) are accessible via a website, allowing individuals to engage in discussions on their EOL preferences and document them while in the community. Residents have also been encouraged to have ACP conversations with their designated family physicians [42]. These efforts to encourage goals-of-care conversations among patients prior to an admission could potentially moderate the use of aggressive care at the EOL for future ESOD cohorts.

Within the hospital, ensuring timely referral and receipt of PC in patients likely to have MC or EC trajectories would allow them to have conversations on EOL care preferences and decisions with the clinical team prior to the final months of life, thereby reducing unnecessary or aggressive care for patients opting for comfort care, improving their quality of life and lowering acute hospital utilisation and costs [43–45]. Specific to the critical care setting, integration of PC can improve quality of EOL care and curb the escalation of EOL cost. PC in the ICU setting has been associated with reductions in ICU and hospital LOS, as well as improvements in family communication and patient quality of life [46]. While half of the patients with EC also received a referral to inpatient PC, it is unclear if the referral had any impact on the decision by the patient or family to receive life-sustaining treatment or be admitted to ICU, which would require further study to ascertain. As access to palliative consult early in an admission has also been demonstrated to reduce overall hospital costs, ensuring patients and their families receive access to a palliative consult at their start of their ICU stay could facilitate the provision of patient-centred care and reduce the cost impact on families by ensuring judicious use of cost-intensive treatments.

Taking a broader perspective, our findings of relatively low healthcare utilization for multiple years before a spike in the final months reflect the lack of alternative avenues for EOL care among most ESOD patients in Singapore during the study period. As with our previous study characterising the cost burden associated with each ESOD [8] patients with AD tended to incur lower costs in the hospital, likely attributable to the presence of community-based dementia care [47, 48]. Access to palliative care through community- or home-based care have been suggested to impact acute healthcare utilization and costs [49]. In a move to expand nationwide access to quality palliative care services in 2023, the Ministry of Health in Singapore announced an expansion of inpatient, home and day hospice capacity and increased subsidies and coverage for palliative services, facilitating receipt of timely PC among patients with life-limiting illnesses. Future work can explore the impact of policy interventions in increasing access and improving quality of PC provision in Singapore.

Our study on five-year HCE trajectories in ESOD adds to the evidence base on the cost burden of this group, and our insights can be informative for several other health systems where a substantial portion of EOL care for ESOD remains sited within the hospital setting. This was also the first study to look at subgroups within ESOD, and we incorporated a validation of the clustering results to ensure reliability in number of subgroups identified. However, this study is not without limitations. As this was a retrospective study, we were only able to describe statistical associations between patient characteristics and likelihood of having a high-cost trajectory. Patients were also classified into diagnosis groups retrospectively, as the definitions for each ESOD group was based on their diagnosis and healthcare utilisation in the last year of life, which cannot be applied directly for prospective identification. We also acknowledge that despite incorporating additional measures such as a recent history of hospitalisations and infections to proxy a diagnosis of advanced disease, some patients may not truly be at the end-stage of the disease. While we focused only on hospital-based cost, with 62% of deaths occurring within hospitals [50], this study would have accounted for the bulk of EOL healthcare resource use. We did not have sufficient information on patients’ disease severity at point of admission, which may account for differences in propensity for EC instead of MC. We also did not have access to patients’ advance care plans to examine if patients with high cost had indicated preferences to persist with curative treatment. However, with the current low nationwide ACP uptake [51], and low palliative referral rates observed in our cohort, we expect that the proportion of patients with completed care plans would likely be too low to influence the cost trajectories observed.

## Conclusion

We highlight the three prominent EOL resource use patterns underlying ESOD, their patient profiles, and risk factors associated with incurring high costs. Our findings could provide evidence to inform the development and targeting of strategies specific to each subgroup. By addressing the gaps and needs for each profile, we can achieve both aims of improving patient care, where patients receive appropriate care in alignment with their needs and preferences along their disease trajectory; and mitigating the cost increase at the EOL, due to judicious use of cost-intensive procedures in patients’ final months of life.

## Electronic supplementary material

Below is the link to the electronic supplementary material.

Supplementary Material 1

Supplementary Material 2

Supplementary Material 3

Supplementary Material 4

Supplementary Material 5

Supplementary Material 6

Supplementary Material 7

Supplementary Material 8

## Data Availability

The datasets generated and/or analysed during the current study are not publicly available due to NHG’s data protection policies to ensure data privacy of patients. Aggregate data may be made available from the corresponding author on reasonable request.
