# Supplementary material for: Identifying clusters of healthcare expenditure trajectories in end-stage organ disease: a retrospective cohort study using linked administrative databases in Singapore
Source: BMC Health Serv Res. 2025 Oct 22;25:1403. doi: 10.1186/s12913-025-13590-z (PMC12548215; doi:10.1186/s12913-025-13590-z)
Supplement: Supplementary file 1 — Supplementary Material 1 [file 12913_2025_13590_MOESM1_ESM.docx]

**Additional File 1.** **Diagnoses described in Kaur *et al***

| **Terminal disease** | **Database criteria** |
| --- | --- |
| **Advanced cancer** | ICD-10-CM   - C77, C78, C79, C80 |
| **Heart failure** | ICD-10-CM   - I50, I11.0, I13.0, I13.2   Additional criteria   - Had two or more hospitalizations in the last 12 months |
| **Respiratory failure** | ICD-10-CM   - J44, J96.1   Additional criteria   - Had two or more hospitalizations in the last 12 months |
| **Kidney failure** | ICD-10-CM   - N18.5 |
| **Severe liver disease** | ICD-10-CM   - K70.4, K71.1, K72.1, K72.9, K76.5, K76.6, K76.7 |
| **Advanced dementia** | ICD-10-CM (Dementia)   - F00, F01, F02, F03, F05.1, G30   Additional criteria  ICD-10-CM (Pneumonia)   - J12, J13, J14, J15, J16, J17, J18   ICD-10-CM (Sepsis)   - A39.2, A39.3, A39.4, A40, A42.7, B00.7, B37.7 - On enteral tube feeding; or - Had pneumonia in the past year prior to death; or - Two or more hospitalizations in the past 12 months for infection or sepsis |
