# Supplementary material for: Identifying clusters of healthcare expenditure trajectories in end-stage organ disease: a retrospective cohort study using linked administrative databases in Singapore
Source: BMC Health Serv Res. 2025 Oct 22;25:1403. doi: 10.1186/s12913-025-13590-z (PMC12548215; doi:10.1186/s12913-025-13590-z)
Supplement: Supplementary file 2 — Supplementary Material 2 [file 12913_2025_13590_MOESM2_ESM.docx]

**Additional File 2. Diagnosis codes used in inclusion criteria**

|  | **ICD-10 code** | **Description** | **Present in data** |
| --- | --- | --- | --- |
| **Heart failure** | I50 | Heart Failure | Y |
|  | I50.0 | Congestive heart failure |  |
|  | I50.1 | Left ventricular failure, unspecified | Y |
|  | I50.2 | Systolic (congestive) heart failure |  |
|  | I50.3 | Diastolic (congestive) heart failure |  |
|  | I50.4 | Combined systolic (congestive) and diastolic (congestive) heart failure |  |
|  | I50.8 | Other heart failure |  |
|  | I50.9 | Heart failure, unspecified | Y |
|  | I11.0 | Hypertensive heart disease with (congestive) heart failure | Y |
|  | I13.0 | Hypertensive heart and renal disease with (congestive) heart failure | Y |
|  | I13.2 | Hypertensive heart and renal disease with both (congestive) heart failure and renal failure | Y |
| **Respiratory failure** | B90.9 | Sequelae of respiratory and unspecified tuberculosis | Y |
|  | I27.23 | Pulmonary hypertension due to lung diseases and hypoxia |  |
|  | I27.81 | Cor pulmonale (chronic) |  |
|  | I27.9 | Pulmonary heart disease, unspecified | Y |
|  | J41 | Simple and mucopurulent chronic bronchitis |  |
|  | J41.0 | Simple chronic bronchitis | Y |
|  | J41.1. | Mucopurulent chronic bronchitis |  |
|  | J41.8 | Mixed simple and mucopurulent chronic bronchitis |  |
|  | J42 | Unspecified chronic bronchitis | Y |
|  | J43 | Emphysema |  |
|  | J43.1 | Panlobular emphysema |  |
|  | J43.2 | Centrilobular emphysema |  |
|  | J43.8 | Other emphysema | Y |
|  | J43.9 | Emphysema, unspecified | Y |
|  | J44.0 | Chronic obstructive pulmonary disease with acute lower respiratory infection | Y |
|  | J44.1 | Chronic obstructive pulmonary disease with (acute) exacerbation | Y |
|  | J44.8 | Other specified chronic obstructive pulmonary disease | Y |
|  | J44.9 | Chronic obstructive pulmonary disease, unspecified | Y |
|  | J47 | Bronchiectasis | Y |
|  | J47.0 | Bronchiectasis with acute lower respiratory infection |  |
|  | J47.1 | Bronchiectasis with (acute) exacerbation |  |
|  | J47.9 | Bronchiectasis, uncomplicated |  |
|  | J60 | Coalworker's pneumoconiosis | Y |
|  | J61 | Pneumoconiosis due to asbestos and other mineral fibers | Y |
|  | J62.8 | Pneumoconiosis due to other dust containing silica | Y |
|  | J63 | Pneumoconiosis due to other inorganic dusts |  |
|  | J64 | Unspecified pneumoconiosis | Y |
|  | J65 | Pneumoconiosis associated with tuberculosis |  |
|  | J67 | Hypersensitivity pneumonitis due to organic dust |  |
|  | J67.0 | Farmer's lung |  |
|  | J67.2 | Bird fancier's lung |  |
|  | J67.8 | Hypersensitivity pneumonitis due to other organic dusts |  |
|  | J67.9 | Hypersensitivity pneumonitis due to unspecified organic dust | Y |
|  | J68.4 | Chronic respiratory conditions due to chemicals, gases, fumes and vapors |  |
|  | J70.3 | Chronic drug-induced interstitial lung disorders |  |
|  | J70.4 | Drug-induced interstitial lung disorders, unspecified | Y |
|  | J84 | Other interstitial pulmonary diseases |  |
|  | J84.1 | Other interstitial pulmonary diseases with fibrosis | Y |
|  | J84.10 | Pulmonary fibrosis, unspecified |  |
|  | J84.11 | Idiopathic interstitial pneumonia |  |
|  | J84.111 | Idiopathic interstitial pneumonia, not otherwise specified |  |
|  | J84.112 | Idiopathic pulmonary fibrosis |  |
|  | J84.113 | Idiopathic non-specific interstitial pneumonitis |  |
|  | J84.114 | Acute interstitial pneumonitis |  |
|  | J84.115 | Respiratory bronchiolitis interstitial lung disease |  |
|  | J84.116 | Cryptogenic organizing pneumonia |  |
|  | J84.117 | Desquamative interstitial pneumonia |  |
|  | J84.17 | Other interstitial pulmonary diseases with fibrosis in diseases classified elsewhere |  |
|  | J84.8 | Other specified interstitial pulmonary diseases | Y |
|  | J84.81 | Lymphangioleiomyomatosis |  |
|  | J84.82 | Adult pulmonary Langerhans cell histiocytosis |  |
|  | J84.89 | Other specified interstitial pulmonary diseases |  |
|  | J84.9 | Interstitial pulmonary disease, unspecified | Y |
|  | J96 | Respiratory failure, not elsewhere classified | Y |
|  | J96.0 | Acute respiratory failure | Y |
|  | J96.1 | Chronic respiratory failure | Y |
|  | J96.10 | Chronic respiratory failure, unspecified whether with hypoxia or hypercapnia |  |
|  | J96.11 | Chronic respiratory failure with hypoxia |  |
|  | J96.12 | Chronic respiratory failure with hypercapnia |  |
|  | J96.2 | Acute and chronic respiratory failure |  |
|  | J96.20 | Acute and chronic respiratory failure, unspecified whether with hypoxia or hypercapnia |  |
|  | J96.21 | Acute and chronic respiratory failure with hypoxia |  |
|  | J96.22 | Acute and chronic respiratory failure with hypercapnia |  |
|  | J96.9 | Respiratory failure, unspecified | Y |
|  | J96.90 | Respiratory failure, unspecified, unspecified whether with hypoxia or hypercapnia |  |
|  | J96.91 | Respiratory failure, unspecified with hypoxia |  |
|  | J96.92 | Respiratory failure, unspecified with hypercapnia |  |
|  | J98.2 | Interstitial emphysema | Y |
|  | M05.1 | Rheumatoid lung disease with rheumatoid arthritis, also includes M05.10 to M05.19 |  |
|  | M05.14 | Rheumatoid lung disease, hand | Y |
|  | M05.17 | Rheumatoid lung disease, ankle and foot | Y |
|  | M05.19 | Rheumatoid lung disease, site unspecified | Y |
|  | M32.13 | Lung involvement in systemic lupus erythematosus |  |
|  | M33.11 | Other dermatomyositis with respiratory involvement |  |
|  | M33.21 | Polymyositis with respiratory involvement |  |
|  | M33.91 | Dermatopolymyositis, unspecified with respiratory involvement |  |
|  | M34.81 | Systemic sclerosis with lung involvement |  |
|  | M35.02 | Sicca syndrome with lung involvement |  |
| **Advanced dementia** | F00 | Dementia in Alzheimer disease | Y |
|  | F00.0 | Dementia in Alzheimer disease with early onset | Y |
|  | F00.1 | Dementia in Alzheimer disease with late onset | Y |
|  | F00.2 | Dementia in Alzheimer disease, atypical or mixed type | Y |
|  | F00.9 | Dementia in Alzheimer disease, unspecified | Y |
|  | F01 | Vascular dementia | Y |
|  | F01.0 | Vascular dementia of acute onset | Y |
|  | F01.1 | Multi-infarct dementia | Y |
|  | F01.2 | Subcortical vascular dementia | Y |
|  | F01.3 | Mixed cortical and subcortical vascular dementia | Y |
|  | F01.8 | Other vascular dementia | Y |
|  | F01.9 | Vascular dementia, unspecified | Y |
|  | F02 | Dementia in other diseases classified elsewhere | Y |
|  | F02.0 | Dementia in Pick disease | Y |
|  | F02.1 | Dementia in Creutzfeldt-Jakob disease | Y |
|  | F02.2 | Dementia in Huntington disease |  |
|  | F02.3 | Dementia in Parkinson disease | Y |
|  | F02.4 | Dementia in human immunodeficiency virus [HIV] disease | Y |
|  | F02.8 | Dementia in other specified diseases classified elsewhere | Y |
|  | F03 | Unspecified dementia | Y |
|  | F05.1 | Delirium superimposed on dementia | Y |
|  | G30 | Alzheimer disease |  |
|  | G30.0 | Alzheimer disease with early onset | Y |
|  | G30.1 | Alzheimer disease with late onset | Y |
|  | G30.8 | Other Alzheimer's disease | Y |
|  | G30.9 | Alzheimer's disease, unspecified | Y |
| **Severe liver disease** | K70.4 | Alcoholic hepatic failure | Y |
|  | K71.1 | Toxic liver disease with hepatic necrosis | Y |
|  | K72.1 | Chronic hepatic failure | Y |
|  | K72.9 | Hepatic failure, unspecified | Y |
|  | K76.5 | Hepatic veno-occlusive disease |  |
|  | K76.6 | Portal hypertension | Y |
|  | K76.7 | Hepatorenal syndrome | Y |
|  | K70.2 | Alcoholic fibrosis and sclerosis of liver | Y |
|  | K70.3 | Alcoholic cirrhosis of liver | Y |
|  | K74 | Fibrosis and cirrhosis of liver | Y |
|  | K74.0 | Hepatic fibrosis | Y |
|  | K74.3 | Primary biliary cirrhosis | Y |
|  | K74.4 | Secondary biliary cirrhosis | Y |
|  | K74.5 | Biliary cirrhosis, unspecified | Y |
|  | K74.6 | Other and unspecified cirrhosis of liver | Y |
| **Kidney failure** | N18.5 | Chronic kidney disease, stage 5 | Y |

|  | **ICD-10 code** | **Description** | **Present in data** |
| --- | --- | --- | --- |
| **Pneumonia** | B052 | Measles complicated by pneumonia |  |
|  | J12 | Viral pneumonia, not elsewhere classified |  |
|  | J12.0 | Adenoviral pneumonia | Y |
|  | J12.1 | Respiratory syncytial virus pneumonia |  |
|  | J12.2 | Parainfluenza virus pneumonia | Y |
|  | J12.3 | Human metapneumovirus pneumonia |  |
|  | J12.8 | Other viral pneumonia | Y |
|  | J12.9 | Viral pneumonia, unspecified | Y |
|  | J13 | Pneumonia due to Stretococcus pneumonia | Y |
|  | J14 | Pneumonia due to Hemophilus influenzae | Y |
|  | J15 | Bacterial pneumonia, not elsewhere classified |  |
|  | J15.0 | Pneumonia due to Klebsiella pneumoniae | Y |
|  | J15.1 | Pneumonia due to Pseudomonas | Y |
|  | J15.2 | Pneumonia due to staphylococcus |  |
|  | J15.3 | Pneumonia due to streptococcus, group B |  |
|  | J15.4 | Pneumonia due to other streptococci | Y |
|  | J15.5 | Pneumonia due to Escherichia coli | Y |
|  | J15.6 | Pneumonia due to other Gram-negative bacteria | Y |
|  | J15.7 | Pneumonia due to Mycoplasma pneumoniae |  |
|  | J15.8 | Pneumonia due to other specified bacteria | Y |
|  | J15.9 | Unspecified bacterial pneumonia | Y |
|  | J16 | Pneumonia due to other infectious organisms, not elsewhere classified |  |
|  | J16.0 | Chlamydial pneumonia |  |
|  | J16.8 | Pneumonia due to other specified infectious organisms |  |
|  | J17 | Pneumonia in diseases classified elsewhere | Y |
|  | J18 | Pneumonia, unspecified organism | Y |
|  | J18.0 | Bronchopneumonia, unspecified organism | Y |
|  | J18.1 | Lobar pneumonia, unspecified organism | Y |
|  | J18.2 | Hypostatic pneumonia, unspecified organism |  |
|  | J18.8 | Other pneumonia, unspecified organism | Y |
|  | J18.9 | Pneumonia, unspecified organism | Y |
|  | J85.1 | Abscess of lung with pneumonia |  |
|  | J85.2 | Abscess of lung without pneumonia |  |
|  | A02.1 | Salmonella sepsis |  |
|  | A04.7 | Enterocolitis due to Clostridium difficile |  |
|  | A19 | Milliary Tuberculosis | Y |
|  | A24 | Glanders and melioidosis |  |
|  | A24.1 | Acute and fulminating melioidosis | Y |
|  | A30 | Leprosy [Hansen's disease] |  |
|  | A31 | Infection due to other mycobacteria | Y |
|  | A39.2 | Acute meningococcaemia |  |
|  | A39.3 | Chronic meningococcaemia |  |
|  | A39.4 | Meningococcaemia, unspecified |  |
|  | A39.4 | Meningococcaemia, unspecified |  |
|  | A40 | Streptococcal sepsis |  |
| **Sepsis** | A40.0 | Sepsis due to Streptococcus, group A |  |
|  | A40.1 | Sepsis due to Streptococcus, group B | Y |
|  | A40.2 | Sepsis due to Streptococcus, group D | Y |
|  | A40.3 | Sepsis due to Streptococcus pneumoniqe |  |
|  | A40.8 | Other streptococcal sepsis | Y |
|  | A40.9 | Streptococcal sepsis, unspecified | Y |
|  | A41 | Other sepsis |  |
|  | A41.0 | Sepsis due to Staphylococcus aureus | Y |
|  | A41.1 | Other sepsis | Y |
|  | A41.2 | Sepsis due to unspecified Staphylococcus |  |
|  | A41.3 | Sepsis due to Haemophilus influenzae |  |
|  | A41.4 | Sepsis due to anaerobes | Y |
|  | A41.5 | Sepsis due to other Gram-negative organisms |  |
|  | A41.50 | Sepsis due to Escherichia coli | Y |
|  | A41.51 | Sepsis due to Pseudomonas | Y |
|  | A41.52 | Sepsis due to Serratia | Y |
|  | A41.58 | Sepsis due to other Gram-negative organisms, NOS | Y |
|  | A41.8 | Other specified sepsis | Y |
|  | A41.80 | Sepsis due to Enterococcus |  |
|  | A41.88 | Other specified sepsis |  |
|  | A41.9 | Sepsis, unspecified, includes: septicaemia | Y |
|  | A42.7 | Actinomycotic sepsis |  |
|  | A49 | Bacterial infection of unspecified site | Y |
|  | A54.8 | Other gonococcal infections |  |
|  | A90 | Dengue fever [classical dengue] |  |
|  | A91 | Dengue hemorrhagic fever |  |
|  | A98 | Other viral hemorrhagic fevers, not elsewhere classified |  |
|  | A99 | Unspecified viral hemorrhagic fever |  |
|  | B00.7 | Disseminated herpesviral disease |  |
|  | B00.9 | Herpesviral infection, unspecified |  |
|  | B01.8 | Varicella with other complications |  |
|  | B01.9 | Varicella without complication |  |
|  | B02.7 | Disseminated zoster |  |
|  | B02.8 | Zoster with other complications |  |
|  | B02.9 | Zoster without complications |  |
|  | B34.9 | Viral infection, unspecified |  |
|  | B37.7 | Candidal sepsis |  |
|  | B95 | Streptococcus, Staphylococcus, and Enterococcus as the cause of diseases classified elsewhere |  |
|  | B95.48 | Other Streptococcus as the cause of diseases clas |  |
|  | B95.6 | S. aureus as the cause of diseases classified else | Y |
|  | B96 | Other bacterial agents as the cause of diseases classified elsewhere | Y |
|  | B96.2 | E. coli as the cause of diseases classified elsewh | Y |
|  | B97 | Viral agents as the cause of diseases classified elsewhere |  |
|  | B99 | Other and unspecified infectious diseases | Y |
|  | D65 | Disseminated intravascular coagulation [defibrination syndrome] |  |
|  | J18.9 | Pneumonia, unspecified organism |  |
|  | J44.0 | Chronic obstructive pulmonary disease with acute l |  |
|  | N39.0 | Urinary tract infection, site not specified |  |
|  | R50 | Fever of other and unknown origin | Y |
|  | T80.2 | Infections following infusion, transfusion and therapeutic injection |  |
|  | T81.4 | Infection following a procedure, not elsewhere classified |  |
|  | T83.8 | Other complications of genitourinary prosthetic devices, implants and grafts |  |
|  | T84.5 | Infection and inflammatory reaction due to internal joint prosthesis |  |
|  | T84.6 | Infection and inflammatory reaction due to internal fixation device [any site] |  |
|  | T84.7 | Infection and inflammatory reaction due to other internal orthopaedic prosthetic devices, implants and grafts |  |
|  | T85.7 | Infection and inflammatory reaction due to other internal prosthetic devices, implants and grafts |  |
|  | Y95 | Nosocomial condition |  |
