# Supplementary material for: Identifying clusters of healthcare expenditure trajectories in end-stage organ disease: a retrospective cohort study using linked administrative databases in Singapore
Source: BMC Health Serv Res. 2025 Oct 22;25:1403. doi: 10.1186/s12913-025-13590-z (PMC12548215; doi:10.1186/s12913-025-13590-z)
Supplement: Supplementary file 3 — Supplementary Material 3 [file 12913_2025_13590_MOESM3_ESM.docx]

**Additional File 3. Characteristics of patients with end-stage organ disease studied**

|  |  | **Total (n=7,154)** |
| --- | --- | --- |
| **ESOD diagnosis** |  | N (%) |
| Advanced dementia |  | 2,970 (41.5%) |
| Kidney failure |  | 2,074 (29.0%) |
| Heart failure |  | 2,435 (34.0%) |
| Respiratory failure |  | 2,075 (29.0%) |
| Severe liver disease |  | 828 (11.6%) |
|  |  |  |
| **Number of ESOD diagnoses** | 1 | 4,668 (65.3%) |
|  | 2 | 1,827 (25.5%) |
|  | 3 | 576 (8.0%) |
|  | 4 | 83 (1.2%) |
|  |  |  |
| **Years from first diagnosis to death** | Median (25^th^ – 75^th^ percentile) | 1.5 (0.3-3.3) |
|  |  |  |
| **Age category** | Below 65 years | 889 (12.4%) |
|  | 65-74 years | 1,244 (17.4%) |
|  | 75-84 years | 2,136 (29.9%) |
|  | 85 years and above | 2,885 (40.3%) |
|  |  |  |
| **Gender** | Female | 3,502 (49.0%) |
|  | Male | 3,652 (51.0%) |
|  |  |  |
| **Ethnicity** | Majority | 5,342 (74.7%) |
|  | Minority | 1,812 (25.3%) |
|  |  |  |
| **Residence type** | Public housing, 1-2-room | 442 (6.2%) |
|  | Public housing, 3-4-room | 3178 (44.4%) |
|  | Public housing, 5-room and larger | 1204 (16.8%) |
|  | Residential nursing home | 1451 (20.3%) |
|  | Private or other housing | 879 (12.3%) |
|  |  |  |
| **Ever referred to inpatient palliative care** |  | 1,758 (24.6%) |

ESOD: end-stage organ disease
