# Supplementary material for: Identifying clusters of healthcare expenditure trajectories in end-stage organ disease: a retrospective cohort study using linked administrative databases in Singapore
Source: BMC Health Serv Res. 2025 Oct 22;25:1403. doi: 10.1186/s12913-025-13590-z (PMC12548215; doi:10.1186/s12913-025-13590-z)
Supplement: Supplementary file 5 — Supplementary Material 5 [file 12913_2025_13590_MOESM5_ESM.docx]

**Additional File 5. Diagnostic plots for sensitivity analyses excluding patients diagnosed in the last month of life, comprising (A) standardized criterion scores across the classification of patient cohort into 3-7 clusters, compared to 2 clusters, and (B) stability of Calinski Harabatz scores over 50 iterations**

| **A** | **B** | |
| --- | --- | --- |
| **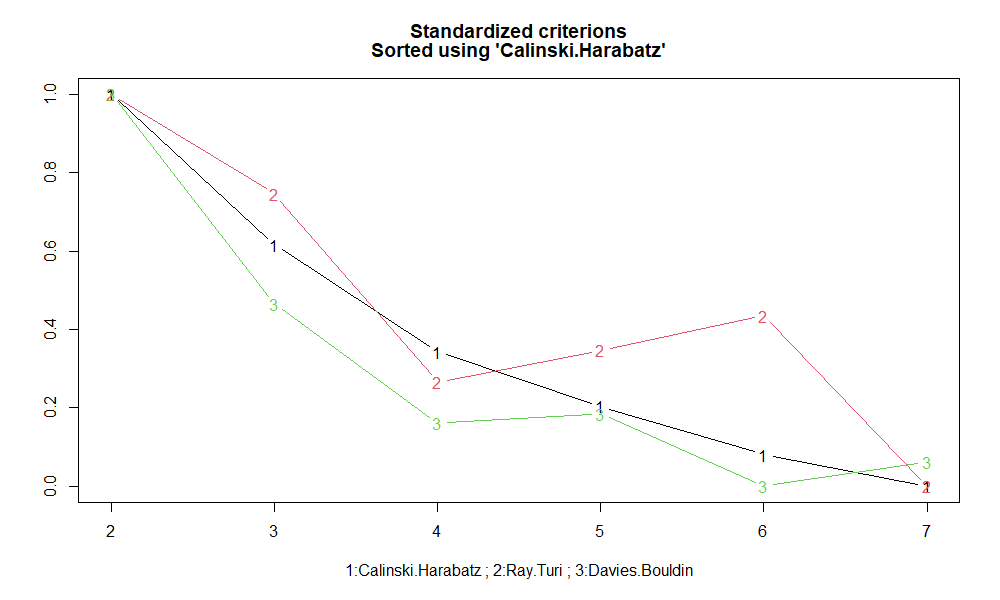** | **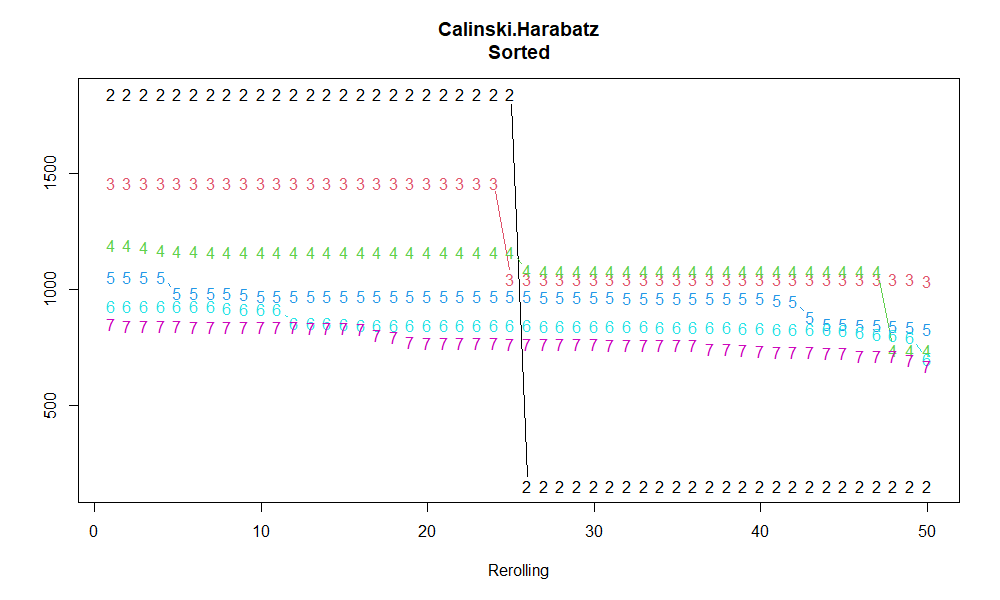** |  |

Panel A illustrates that in classifying the patient cohort into 2-7 groups, the 2-group approach has the highest standardized criterion scores, followed by the 3-group approach. Panel B illustrates the relative instability of the 2-group approach as scores decrease to near zero after 20 iterations, demonstrating that the 3-group approach has the highest and most stable criterion scores.
