# Supplementary material for: Identifying clusters of healthcare expenditure trajectories in end-stage organ disease: a retrospective cohort study using linked administrative databases in Singapore
Source: BMC Health Serv Res. 2025 Oct 22;25:1403. doi: 10.1186/s12913-025-13590-z (PMC12548215; doi:10.1186/s12913-025-13590-z)
Supplement: Supplementary file 6 — Supplementary Material 6 [file 12913_2025_13590_MOESM6_ESM.docx]

**Additional File 6. Healthcare expenditure in last 3 months, 1 year and 5 years of life**

|  | **Consistently low-cost**  **(n=5,756)** | **Moderately high cost near death (n=1,283)** | **Escalating cost near death**  **(n=115)** |  | **Consistently low-cost**  **(n=5,756)** | | **Moderately high cost near death (n=1,283)** | **Escalating cost near death**  **(n=115)** |
| --- | --- | --- | --- | --- | --- | --- | --- | --- |
| **Last 3 months of life** | **Mean (SD)** | | |  | **Median (Q1-Q3)** | | | |
| Mean HCE | £ 6,715  (£ 5,433) | £ 31,002  (£ 11,216) | £ 104,626  (£ 48,618) |  | £ 5,707  (£ 2,025-£ 10,657) | £ 27,836  (£ 22,533-£ 36,910) | | £ 84,663  (£ 75,086-£ 116,046) |
|  |  |  |  |  |  |  | |  |
| Mean HCE by setting |  |  |  |  |  |  | |  |
| Inpatient admissions | £ 6,110  (£ 5,287) | £ 30,142  (£ 11,350) | £ 104,076  (£ 48,734) |  | £ 5,017  (£ 1,408-£ 9,958) | £ 26,816  (£ 21,847-£ 36,036) | | £ 84,150  (£ 74,452-£ 115,910) |
| ED attendances | £ 384  (£ 326) | £ 556  (£ 445) | £ 365  (£ 381) |  | £ 318  (£ 174-£ 541) | £ 472  (£ 271-£ 739) | | £ 291  (£ 0-£ 503) |
| SOC visits | £ 197  (£ 356) | £ 263  (£ 700) | £ 150  (£ 376) |  | £ 41  (£ 0-£ 277) | £ 77  (£ 0-£ 319) | | £ 0  (£ 0-£ 139) |
| Day procedures | £ 23  (£ 217) | £ 41  (£ 298) | £ 35  (£ 237) |  | £ 0  (£ 0-£ 0) | £ 0  (£ 0-£ 0) | | £ 0  (£ 0-£ 0) |
|  |  | | |  |  | | | |
| **Last 1 year of life** | **Mean (SD)** | | |  | **Median (Q1-Q3)** | | | |
| Mean HCE | £ 14,692  (£ 11,354) | £ 46,466  (£ 24,384) | £ 119,676  (£ 56,169) |  | £ 12,143  (£ 6,680-£ 19,446) | | £ 40,039  (£ 29,740-£ 56,386) | £ 98,590  (£ 85,215-£ 130,117) |
|  |  |  |  |  |  | |  |  |
| Mean HCE by setting |  |  |  |  |  | |  |  |
| Inpatient admissions | £ 12,960  (£ 10,816) | £ 43,753  (£ 23,908) | £ 117,213  (£ 56,344) |  | £ 10,671  (£ 5,400-£ 17,485) | | £ 37,185  (£ 27,408-£ 53,109) | £ 95,904  (£ 83,839-£ 128,732) |
| ED attendances | £ 816  (£ 710) | £ 1,209  (£ 1,024) | £ 911  (£ 620) |  | £ 649  (£ 377-£ 1,053) | | £ 972  (£ 599-£ 1,490) | £ 704  (£ 435-£ 1,253) |
| SOC visits | £ 813  (£ 1,157) | £ 1,296  (£ 2,287) | £ 1,146  (£ 1,322) |  | £ 411  (£ 0-£ 1,209) | | £ 882  (£ 190-£ 1,816) | £ 719  (£ 121-£ 1,513) |
| Day procedures | £ 103  (£ 537) | £ 208  (£ 745) | £ 405  (£ 1,684) |  | £ 0  (£ 0-£ 0) | | £ 0  (£ 0-£ 0) | £ 0  (£ 0-£ 0) |
|  |  |  |  |  |  | |  |  |
| **Last 5 years of life** | **Mean (SD)** | | |  | **Median (Q1-Q3)** | | | |
| Mean HCE | £ 32,519  (£ 27,366) | £ 73,090  (£ 46,338) | £ 148,472  (£ 75,022) |  | £ 25,701  (£ 14,840-£ 41,588) | | £ 60,118  (£ 42,147-£ 89,918) | £ 129,027  (£ 98,732-£ 165,727) |
|  |  |  |  |  |  | |  |  |
| Mean HCE by setting |  |  |  |  |  | |  |  |
| Inpatient admissions | £ 26,829  (£ 24,182) | £ 64,297  (£ 42,462) | £ 139,336  (£ 72,414) |  | £ 20,514  (£ 11,469-£ 34,420) | | £ 52,553  (£ 36,051-£ 79,075) | £ 122,755  (£ 92,774-£ 155,769) |
| ED attendances | £ 1,761  (£ 1,792) | £ 2,355  (£ 2,169) | £ 1,888  (£ 1,548) |  | £ 1,309  (£ 757-£ 2,173) | | £ 1,716  (£ 1,079-£ 2,857) | £ 1,359  (£ 725-£ 2,641) |
| SOC visits | £ 3,351  (£ 4,774) | £ 5,369  (£ 7,569) | £ 5,604  (£ 6,133) |  | £ 1,741  (£ 325-£ 4,748) | | £ 3,544  (£ 859-£ 8,009) | £ 3,812  (£ 678-£ 8,528) |
| Day procedures | £ 577  (£ 1,597) | £ 1,070  (£ 2,177) | £ 1,644  (£ 3,146) |  | £ 0  (£ 0-£ 197) | | £ 0  (£ 0-£ 1,378) | £ 0  (£ 0-£ 1,921) |

ED: Emergency Department; HCE: Healthcare expenditure; Q1: 1^st^ quartile; Q3: 3^rd^ quartile; SD: standard deviation; SOC: Specialist outpatient clinic
