# Supplementary material for: Identifying clusters of healthcare expenditure trajectories in end-stage organ disease: a retrospective cohort study using linked administrative databases in Singapore
Source: BMC Health Serv Res. 2025 Oct 22;25:1403. doi: 10.1186/s12913-025-13590-z (PMC12548215; doi:10.1186/s12913-025-13590-z)
Supplement: Supplementary file 7 — Supplementary Material 7 [file 12913_2025_13590_MOESM7_ESM.docx]

**Additional File 7. Healthcare expenditure trajectories over the final five years of life, excluding the final quarter**
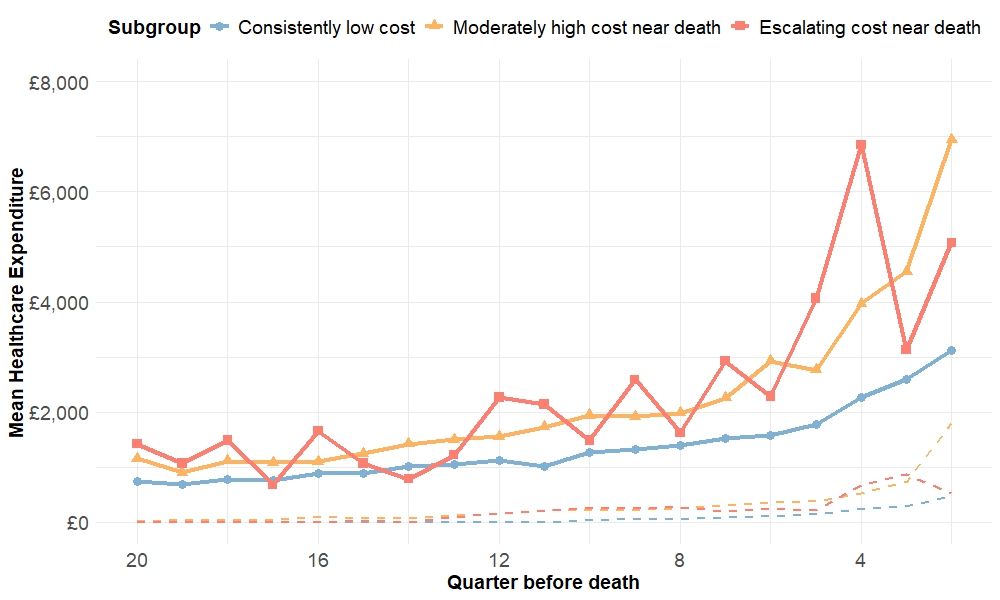


Solid lines reflect mean quarterly healthcare expenditure for each subgroup, while dashed lines reflect median values.
