# Supplementary material for: Identifying clusters of healthcare expenditure trajectories in end-stage organ disease: a retrospective cohort study using linked administrative databases in Singapore
Source: BMC Health Serv Res. 2025 Oct 22;25:1403. doi: 10.1186/s12913-025-13590-z (PMC12548215; doi:10.1186/s12913-025-13590-z)
Supplement: Supplementary file 8 — Supplementary Material 8 [file 12913_2025_13590_MOESM8_ESM.docx]

**Additional File 8. Healthcare utilisation in last 3 months, 1 year and 5 years of life**

|  | **Consistently low-cost**  **(n=5,756)** | **Moderately high cost near death (n=1,283)** | **Escalating cost near death**  **(n=115)** |  | **Consistently low-cost**  **(n=5,756)** | | **Moderately high cost near death (n=1,283)** | **Escalating cost near death**  **(n=115)** |
| --- | --- | --- | --- | --- | --- | --- | --- | --- |
| **Last 3 months of life** | **Mean (SD)** | | |  | **Median (Q1-Q3)** | | | |
| Number of inpatient admissions | 1.3 (1.0) | 2.4 (1.4) | 1.8 (0.9) |  | 1 (1-2) | 2 (1-3) | | 2 (1-2) |
| Admission to intensive care unit | 0.1 (0.3) | 0.4 (0.5) | 0.9 (0.5) |  | 0 (0-0) | 0 (0-1) | | 1 (1-1) |
| Admission to high-dependency unit | 0.1 (0.3) | 0.4 (0.6) | 0.9 (0.6) |  | 0 (0-0) | 0 (0-1) | | 1 (1-1) |
| Inpatient length of stay (days) | 13.4 (12.4) | 50.2 (26.0) | 104.0 (57.3) |  | 11 (3-21) | 47 (32-63) | | 92 (65.5-132) |
| Number of ED attendances | 1.4 (1.1) | 2.1 (1.5) | 1.3 (1.1) |  | 1 (1-2) | 2 (1-3) | | 1 (0-2) |
| Number of SOC visits | 2.3 (3.6) | 2.9 (3.8) | 1.7 (4.1) |  | 1 (0-3) | 2 (0-4) | | 0 (0-2) |
| Number of day procedures | 0.03 (0.20) | 0.04 (0.23) | 0.03 (0.18) |  | 0 (0-0) | 0 (0-0) | | 0 (0-0) |
|  |  | | |  |  | | | |
| **Last 1 year of life** | **Mean (SD)** | | |  | **Median (Q1-Q3)** | | | |
| Number of inpatient admissions | 2.8 (2.2) | 4.6 (3.6) | 3.4 (1.9) |  | 2 (1-4) | | 4 (2-6) | 3 (2-4) |
| Admission to intensive care unit | 0.1 (0.3) | 0.5 (0.6) | 1.0 (0.6) |  | 0 (0-0) | | 0 (0-1) | 1 (1-1) |
| Admission to high-dependency unit | 0.2 (0.4) | 0.6 (0.8) | 1.1 (0.7) |  | 0 (0-0) | | 0 (0-1) | 1 (1-1) |
| Inpatient length of stay (days) | 29.2 (25.5) | 77 (51.6) | 125 (69.2) |  | 23 (11-40) | | 67 (44-97.5) | 114 (75.5-163) |
| Number of ED attendances | 3.1 (2.6) | 4.6 (3.6) | 3.4 (2.3) |  | 2 (1-4) | | 4 (2-6) | 3 (2-4) |
| Number of SOC visits | 8.4 (10.4) | 12.7 (13.1) | 11 (11.9) |  | 5 (1-12) | | 9 (3-18) | 7 (2-14.5) |
| Number of day procedures | 0.1 (0.6) | 0.2 (0.7) | 0.3 (0.8) |  | 0 (0-0) | | 0 (0-0) | 0 (0-0) |
|  |  |  |  |  |  | |  |  |
| **Last 5 years of life** | **Mean (SD)** | | |  | **Median (Q1-Q3)** | | | |
| Number of inpatient admissions | 6.3 (5.9) | 9.1 (7.7) | 7.3 (6.0) |  | 5 (3-8) | | 7 (4-11) | 5 (3-11) |
| Admission to intensive care unit | 0.2 (0.5) | 0.6 (0.8) | 1.1 (0.8) |  | 0 (0-0) | | 0 (0-1) | 1 (1-1) |
| Admission to high-dependency unit | 0.4 (0.7) | 1.0 (1.2) | 1.4 (1.0) |  | 0 (0-1) | | 1 (0-1) | 1 (1-2) |
| Inpatient length of stay (days) | 62.1 (56.8) | 119 (87.9) | 163 (104) |  | 47 (25-81) | | 98 (60.5-156) | 146 (87-217) |
| Number of ED attendances | 7.3 (7.7) | 10 (8.9) | 8.1 (7.0) |  | 5 (3-9) | | 8 (4-12) | 5 (3-11) |
| Number of SOC visits | 32.3 (38.0) | 51.5 (53.6) | 48.9 (51.4) |  | 20 (5-46) | | 37 (11-75.5) | 32 (9.5-69) |
| Number of day procedures | 0.6 (1.8) | 1.2 (2.6) | 1.2 (1.8) |  | 0 (0-1) | | 0 (0-1) | 0 (0-2) |

ED: Emergency Department; HCE: healthcare expenditure; Q1: 1^st^ quartile; Q3: 3^rd^ quartile; SD: standard deviation; SOC: specialist outpatient clinic
